# Supplementary material for: Functional biodiversity loss along natural CO2 gradients
Source: Nat Commun. 2018 Dec 11;9:5149. doi: 10.1038/s41467-018-07592-1 (PMC6288110; doi:10.1038/s41467-018-07592-1)
Supplement: Supplementary file 1 — Supplementary Information [file 41467_2018_7592_MOESM1_ESM.pdf]

Functional biodiversity loss along natural CO_2 gradients

Teixidó et al.

Supplementary Information

Supplementary Figure 1. Intersection of the three functional volumes among pH zones.

Supplementary Figure 2. Null model of functional richness (functional volume) among pH zones.

Supplementary Figure 3. Sensitivity analyses based on a decreased number of categories for each trait.

Supplementary Figure 4. Functional redundancy among pH zones.

Supplementary Figure 5. Vulnerability of FEs among pH zones.

Supplementary Figure 6. Distribution of functional trait categories across the functional space

Supplementary Figure 7. Analysis based on data from north and south venting sampling sites and a decreased number of categories for each trait.

Supplementary Table 1. Macroalgae and invertebrate species identified. Values are mean ± SD of % cover. 24 quadrats of 25 cm*25 cm were sampled for each pH conditions.

Supplementary Table 2. Description of the 15 traits used to measure functional diversity of benthic species based on Text S1.

Supplementary Table 3. Taxonomic and functional β-diversity comparisons among pH zones

Supplementary Table 4. Description of the decreased number of categories to calculate the sensitivity analyses.

Supplementary Table 5. Relative abundance (total sum) of functional trait categories (%) among pH zones.

Supplementary Note 1. Ecological relevance of functional traits

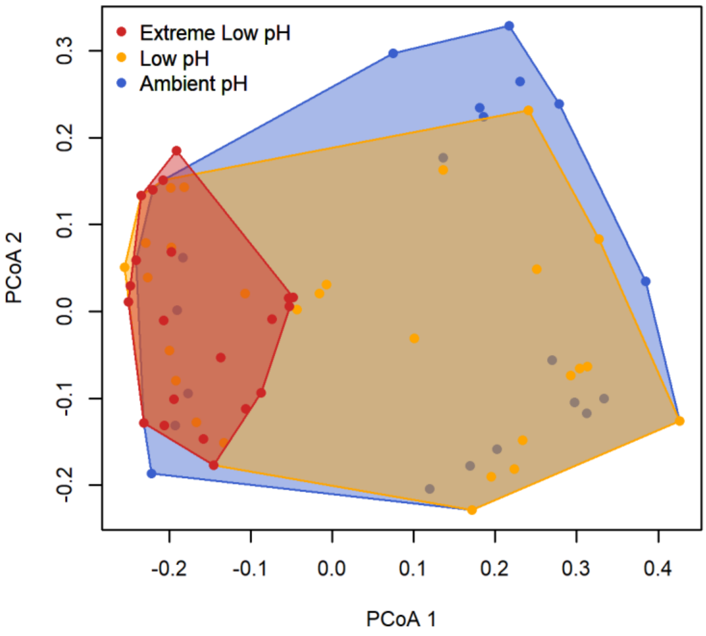

Supplementary Figure 1. Intersection of the three functional volumes among pH zones. Functional β-diversity equals zero when the portions of the functional space filled by species assemblages are perfectly overlapping, and equals unity when assemblages do not intersect in that functional space. Functional β-diversity was high in ambient - extreme low pH (98%) and low - extreme low pH (97%) (Table S3), whereas it showed low values in ambient and low pH zones (17%) with high overlap in the functional space and conservation of functions. In addition, 95% and 92% of the functional volume of low and extreme low pH zones were nested within the volume of ambient pH zones, respectively. 97% of the volume of extreme low pH zone was also nested within the volume of low pH zones. Overall, this means that the trait values of species occurring in acidified conditions represent a subset of trait values found in the ambient pH zones, indicating environmental filtering caused by decreasing pH. Number of species = 72, number of FEs = 68.

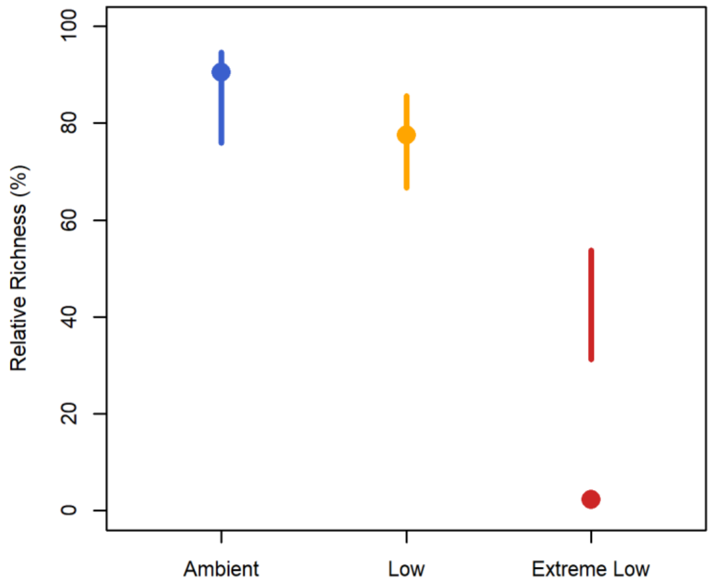

Supplementary Figure 2. Null model of functional richness (functional volume) among pH zones. Points indicate the observed values of functional richness whereas bars represent the 95% confidence interval of expected values under a null model simulating a random sorting of species from the total pool of functional entities while keeping constant the observed local number of species. If the points are within the bars, this means that the functional richness does not deviate from a random expectation. As the random selection is done from the total pool (of which the ambient pH zone participates greatly), it is consistent that the ambient pH zone falls within the random scenario. However, for the extreme low pH zone, functional richness is much smaller than expected by chances as the mean observed value is lower than the confidence interval of expected values indicating a drastic selection of a limited combination of traits (i.e. environmental filtering). Number of species = 72, number of FEs = 68.

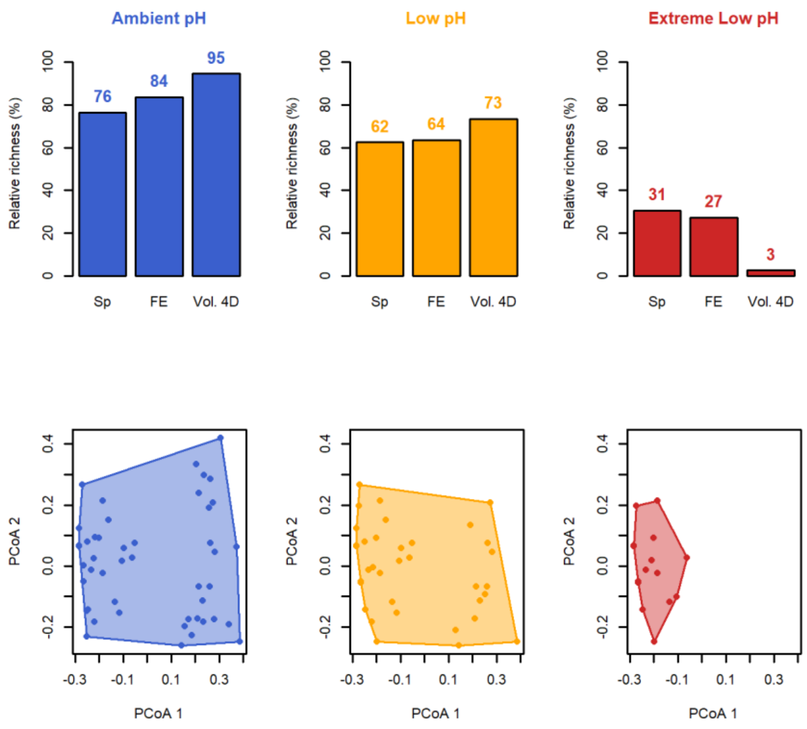

Supplementary Figure 3. Sensitivity analyses based on a decreased number of categories for each trait. We tested whether functional diversity estimates were robust to the resolution of the categorization of functional traits. We reduced the number of categories for each trait (See Table S4 for the description of the reduced number of categories) and re-ran all the analyses. The sensitivity analyses indicated no major changes compared to the results obtained with the finer categorization (Main text, Figure 1). Number of species = 72, number of FEs = 32.

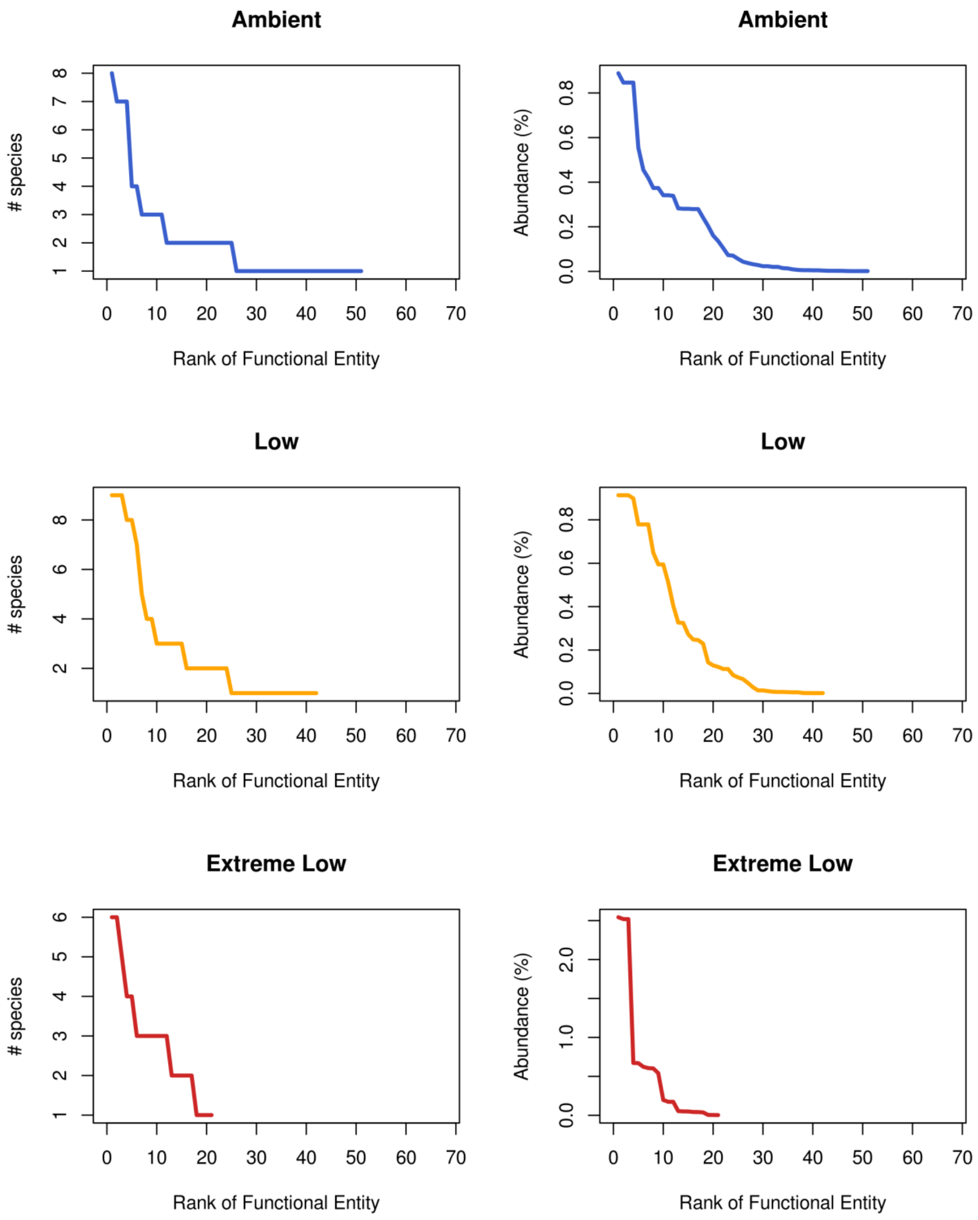

Supplementary Figure 4. Functional redundancy among the pH zones. The distribution of the number of benthic species and its abundance into functional entities (FEs) within a fixed radius (k=1%) is shown for each pH zone. The assumption is that similar species (e.g. closer species) may have similar functions. Under this assumption, high redundancies of several entities are found in the ambient pH zones and this is particularly remarkable in terms of abundance. Abundance is disproportionately packed into a few FEs in the extreme low pH zones. Loss of FE richness is also evident on the X axis. Number of species = 72, number of FEs = 68.

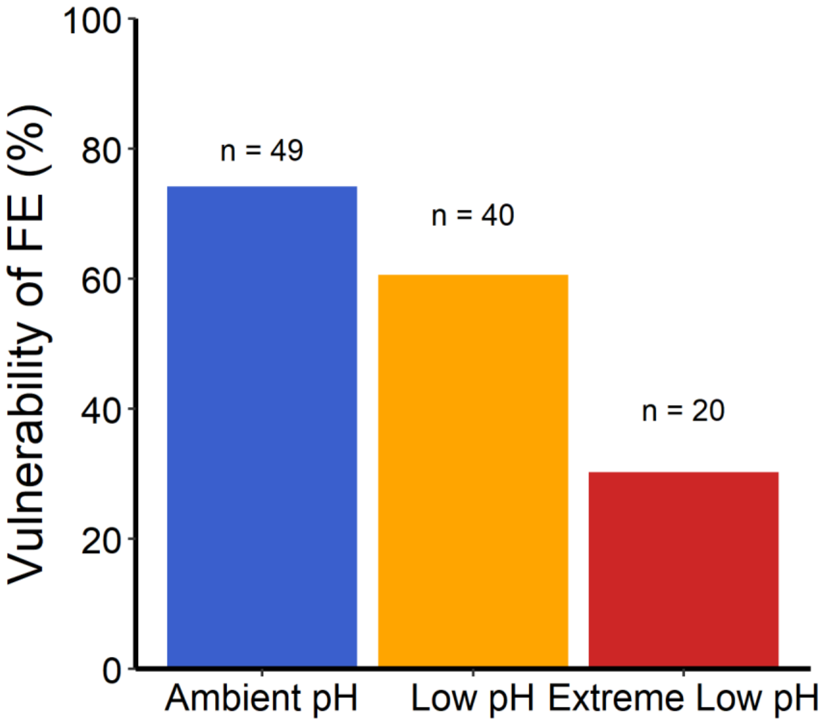

Supplementary Figure 5. Vulnerability of FEs among the pH zones. Vulnerability is expressed as the proportion of FE in an assemblage that had redundancy of 1 (i.e. the proportion of FEs having only one species within the k radius in the functional space). We characterized the traits of 72 benthic species, which resulted in 68 unique trait combinations or functional entities (FE). Accordingly, 66 FEs were classified as vulnerable with having only one species: n=49 Ambient pH, n= 40 Low pH, n= 20 Extreme low pH.

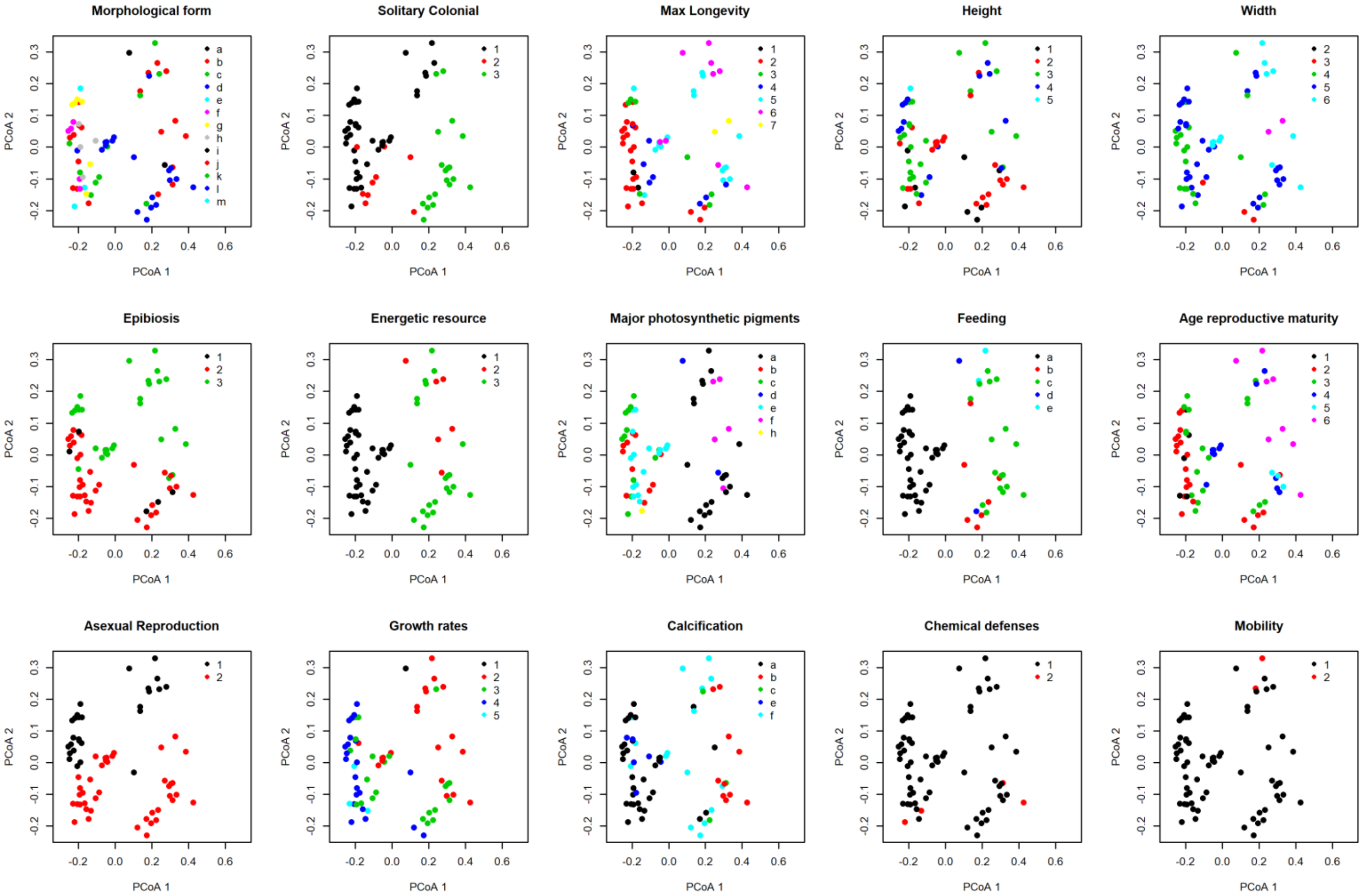

Supplementary Figure 6. Distribution of functional trait categories across the functional space. See Table S2 for trait category descriptions and codes. Species (n=72), Traits (n= 15), Trait categories (n= 73), FEs (n= 68)

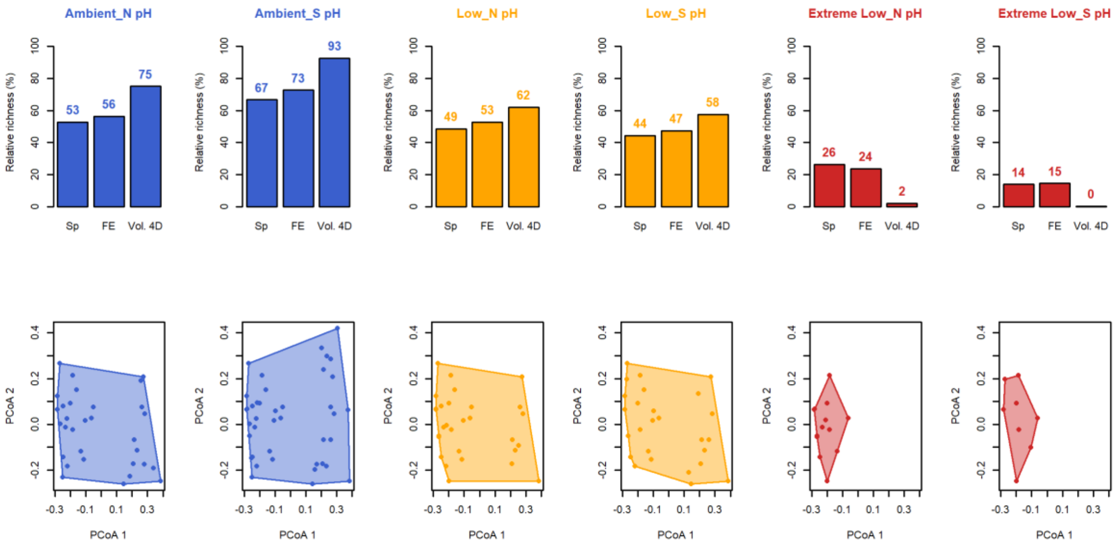

Supplementary Figure 7. Analysis based on data from north and south venting sampling sites and a decreased number of categories for each trait. Before pooling the data from both sides, we performed a set of preliminary analyses to test for statistical differences between north and south sides by using non-parametric analysis of variance PERMANOVA with side and pH as fixed factors. These preliminary analyses did not reveal any significant differences when considering side as a factor (Pseudo F= 1.4, p>0.05 for species; Pseudo-F= 2.8, p>0.05 for FEs;). Number of species = 72, number of FEs = 32, number of replicates = 12 quadrats*2sites*3 pH zones (total number of quadrats = 72).

Supplementary Table 1. **Macroalgae and invertebrate species identified.** Values are mean ± SD of % cover. 24 quadrats of 25 cm* 25 cm were sampled for each pH condition.

Taxa	Ambient pH	Low pH	Extreme Low pH
**Phaeophyceae**			
Aglaozonia cf. *melanoidea*	-	-	0.6 ± 1.5
Cladostephus hirsutus	-	0.1 ± 0.4	0.8 ± 2.2
Colpomenia sinuosa	-	0.1 ± 0.3	-
Dictyota sp.	10.3 ± 10.9	8.6 ± 9.3	14.5 ± 14.2
Dictyota dichotoma v. intricata	1.7 ± 4.3	3.6 ± 4.2	0.3 ± 0.9
Dictyota fasciola	0.1 ± 0.4	0.8 ± 1.4	-
Dictyota spiralis	1.1 ± 1.9	0.3 ± 0.9	-
Halopteris filicina	1.3 ± 4.9	2.4 ± 5.8	0.6 ± 2.5
Halopteris scoparia	5.4 ± 7.0	16.2 ± 8.9	2.7 ± 7.4
Lobophora variegata	0.4 ± 1.1	-	-
*Padina pavonica**	2.3 ± 2.0	0.4 ± 0.7	-
Pseudolithoderma adriaticum	0.6 ± 1.8	0.2 ± 0.6	12.4 ± 13.4
Sargassum vulgare	-	-	0.6 ± 1.7
Sphacelaria cirrosa	2.7 ± 3.2	3.5 ± 3.5	0.5 ± 1.6
Taonia atomaria	-	0.3 ± 0.9	-
**Rhodophyta**			
Acrosorium venulosum	0.04 ± 0.2	-	-
*Amphiroa cryptarthrodia**	0.09 ± 0.3	-	-
*Amphiroa rigida**	0.8 ± 1.6	1.2 ± 3.2	-
*Corallina elongata**	9.5 ± 10.8	3.3 ± 3.9	-
Cryptonemia lomation	-	0.04 ± 0.2	-
Ethelia van bosseae	-	-	5.7 ± 8.6
Gelidium pusillum	-	-	0.4 ± 1.3
*Haliptilon virgatum**	1.1 ± 1.9	-	-
Hildenbrandia crouaniorum	3.3 ± 3.7	12.1 ± 7.1	42.3 ± 20.1
*Hydrolithon farinosum**	2.5 ± 3.9	0.14 ± 0.7	-
*Jania rubens**	1.6 ± 3.1	-	-
*Neogoniolithon brassica-florida**	7.6 ± 8.3	2.5 ± 5.9	-
*Peyssonnelia rosa marina**	1.4 ± 3.6	0.2 ± 0.6	-
Peyssonnelia squamaria	6.5 ± 7.4	6.5 ± 5.2	-
Rhodophyllis divaricata	-	0.04 ± 0.2	0.1 ± 0.5
Rhodymenia ardissonei	0.3 ± 1.3	0.3 ± 1.0	1.2 ± 2.8
**Chlorophyta**			
*Acetabularia acetabulum**	1.4 ± 3.2	0.7 ± 1.1	-
Bryopsis duplex	0.03 ± 0.2	0.04 ± 0.2	-
Caulerpa cylindracea	1.7 ± 4.0	2.9 ± 4.9	-
Cladophora aff. coelothrix	3.6 ± 6.6	4.2 ± 6.4	0.4 ± 1.6
Cladophora coelothrix	5.8 ± 10.9	1.8 ± 3.5	-
Cladophora prolifera	4.8 ± 6.4	7.6 ± 5.9	1.2 ± 2.6
Flabellia petiolata	8.1 ± 8.0	5.9 ± 5.4	1.3 ± 3.5
Halicystis parvula	-	-	0.06 ± 0.3
*Halimeda tuna**	0.07 ± 0.3	0.04 ± 0.2	-
*Parvocaulis parvulus**	0.07 ± 0.2	-	-
Pedobesia lamourouxii	-	0.04 ± 0.2	0.05 ± 0.2
Valonia utricularis	-	-	0.08 ± 0.4

Taxa	Ambient pH	Low pH	Extreme Low pH
**Turf**	-	0.05 ± 0.3	14.1 ± 15.5
**Porifera**			
Cacospongia mollior	0.1 ± 0.6	-	-
Chondrosia reniformis	0.4 ± 0.7	0.2 ± 0.6	-
Cliona viridis	0.2 ± 0.4	-	-
Crambe crambe	1.8 ± 3.7	3.2 ± 4.5	-
Haliclona mediterranea	-	0.1 ± 0.2	-
Hymedesmia versicolor	0.09 ± 0.4	-	-
Ircinia dendroides	0.06 ± 0.3	-	-
Ircinia fasciculata	-	0.04 ± 0.2	-
Myxilla rosacea	0.4 ± 1.8	-	-
Petrosia ficiformis	-	0.04 ± 0.2	-
Terpios fugax	0.04 ± 0.2	-	-
**Cnidaria**			
*Balanophyllia europaea**	0.04 ± 0.2	-	-
Stephanoscyphus sp.	0.1 ± 0.6	-	-
**Bryozoa**			
Celleporina sp.*	0.2 ± 0.7	1.6 ± 3.1	-
*Patinella radiata**	0.4 ± 0.2	0.1 ± 0.4	-
*Schizoporella longicornis**	0.7 ± 2.5	0.3 ± 0.9	-
*Watersipora subovoidea**	-	0.4 ± 0.2	-
**Polychaeta**			
*Salmacina dysteri**	0.4 ± 1.0	-	-
Spirorbinae gen. sp.*	4.8 ± 7.2	5.9 ± 6.8	-
**Mollusca**			
*Arca noae**	0.04 ± 0.2	-	-
*Patella coerulea**	0.04 ± 0.12	-	-
**Echinodermata**			
*Paracentrotus lividus**	0.06 ± 0.2	-	-
**Crustacea**			
*Perforatus perforatus**	3.3 ± 6.0	1.7 ± 2.7	-
**Tunicata**			
Ascidia mentula	0.04 ± 0.2	-	-
*Cystodytes dellechiajei**	0.2 ± 0.5	0.07 ± 0.2	-
Didemnum sp.*	0.7 ± 1.7	0.3 ± 0.8	-
Diplosoma spongiforme	0.09 ± 0.5	-	-
*Pyura dura**	0.03 ± 0.2	-	-

* Denotes species with calcareous structures, ranging from calcareous spicules to continuous carbonate shells and skeletons. See Table S2 for calcification categories

Supplementary Table 2. Description of the 15 traits used to measure functional diversity of benthic species based on Supplementary Note 1. N= number of categories. Genera and species in parenthesis are exhaustive examples of benthic organisms but not all of them necessary found at the Castello study site.

Trait	Trait Type	N	Categories
1) Morphological form	Categorical	13	a) Boring (e.g. *Cliona*)<br>b) Filaments (e.g. *Ceramium*, *Cladophora*)<br>c) Stolonial (e.g. *Caulerpa, Halimeda, Flabellia*)<br>d) Encrusting (e.g. *Palmophyllum, Pseudolithoderma, Mesophyllum, Lithophyllum, Crambe, Spirastrella*)<br>e) Encrusting, leaf-like, with blades (e.g. *Peyssonnelia*)<br>f) Foliose erect thallus, Sheets/blades (e.g. *Dictyota, Ulva, Padina*)<br>g) Coarsely branched (e.g. *Gelidium, Palisada, Sphaerococcus*)<br>h) Articulated (e.g. *Corallina, Haliptilon, Jania*)<br>i) Cup-like (e.g. *Leptopsammia, Caryophyllia*)<br>j) Massive encrusting (height< radius) (e.g. *Chondrosia, Petrosia*)<br>k) Massive hemispheric (height= radius) (e.g. *Codium bursa, Colpomenia, Agelas oroides, Ircinia oros*)<br>l) Massive-erect (height> radius (e.g. *Halocynthia*)<br>m) Tree-like (e.g. *Sargassum, Cystoseira, Laminaria, Axinella polypoides, Paramuricea, Myriapora truncata*)
2) Solitary-Colonial	Ordinal	3	1) Solitary (e.g. *Lithophyllum, Serpula vermicularis, Halocynthia*, solitary corals)<br>2) Gregarious (e.g. *Laminaria, Halimeda*)<br>3) Colonial (e.g. *Palmophyllum crassum*, Porifera, colonial corals, gorgonians, Bryozoa, colonial Tunicata…)
3) Maximum longevity	Ordinal	7	1) weeks (e.g. *Cladophora vagabunda, Acinetospora crinita*)<br>2) 3-11 months (e.g. *Dictyota, Haliptilon*)<br>3) 1 year (e.g. *Laurencia, Sphaerococcus, Halopteris scoparia*)<br>4) 2 years (e.g. *Cystoseira compressa, Corallina, Reteporella*)<br>5) 5 years (e.g. *Cystoseira balearica, Halimeda*)<br>6) 10 years -20 years (e.g. *Pentapora, Schizotheca, Paracentrotus*)<br>7) > 20 years (e.g. *Cystoseira zosteroides*, Massive big sponges, *Paramuricea clavata*)
4) Size (Height)	Ordinal	6	1) up to 1 mm (e.g. *Stylonema, Erythrocladia, Acrochaetium*)<br>2) 1-10 mm (e.g. *Palmophyllum, Nemoderma, Lejolisia*)<br>3) 10-50 mm (e.g. *Gelidium, Chondracanthus, Plocamium*)<br>4) 50-200 mm (e.g. *Halopteris, Padina, Cladostephus*)<br>5) 200-500 mm (e.g. *Dictyopteris, Cystoseira balearica*)<br>6) >500 mm (e.g. *Laminaria, Cystoseira spinosa*)
5) Size (Maximum width)	Ordinal	6	1) up to 0.1 mm (e.g. *Acrochaetium, Callithamniella*)<br>2) 0.1-1 mm (e.g. *Chaetomorpha crassa, Vickersia*)<br>3) 1-10 mm (e.g. *Hydrolithon farinosum, Pedobesia*)<br>4) 10-50 mm (e.g. *Halimeda, Peyssonnelia squamaria*)<br>5) 50-200 mm (e.g. *Lithophyllum incrustans, Neogoniolithon*)<br>6) >200 mm (e.g. *Sarcotragus spinosula, Lithophyllum byssoides*)
6) Epibiosis	Ordinal	3	1) Obligate (e.g. *Amphiroa, Phyllariopsis, Salmacina*)<br>2) Facultative (e.g. *Dictyota, Sphacelaria*)<br>3) Never (e.g. *Pseudolithoderma, Cystoseira spinosa, Paramuricea*)

**Trait**	**Trait Type**	**Nº**	**Categories**
7) Energetic resource (ordinal)	Ordinal	3	1) Photosynthetic autotroph (e.g. algae)<br>2) Photo-heterotroph (e.g. *Cladocora caespitosa*, *Eunicella singularis*, *Chondrilla nucula*)<br>3) Heterotroph (e.g. most invertebrates)
8) Major photosynthetic pigments	Categorical	8	a) No (e.g. most of invertebrates*)*<br>b) Chl a, Chl b, Beta-carotene, Xanthophyll (e.g. green algae)<br>c) Chl a, Xanthophyll /Fucoxanthin, Chl c1+c2 (e.g. brown algae)<br>d) Chla, Chlorophyll c2, Peridinin (e.g. dinoflagellates present in *Cladocora caespitosa*)<br>e) Chl a, Phycocyanin, Phycoerythrin (e.g. red algae)<br>f) Chla, Phycocyanin (Cyanobacteria) (e.g. present in *Petrosia*, *Chondrilla nucula*)<br>g) Bacteriochlorophyll<br>h) Mixture of a), b),c), e) (e.g. turf)
9) Feeding	Categorical	6	a) No (autotroph) (e.g. algae)<br>b) Active filter feeders with cilia (e.g. bryozoans, sabellids)<br>c) Active filter feeders by pumping (e.g. sponges, tunicates, bivalves)<br>d) Passive filter feeders (e.g. cnidarians)<br>e) Herbivores/Grazers (e.g. sea urchins: *Arbacia*, *Paracentrotus*; *Patella*)<br>f) Carnivores (e.g. *Echinaster, Marthasterias, Stramonita, Hexaplex*)
10) Age at reproductive maturity	Ordinal	7	1) weeks (e.g. *Cladophora vagabunda*, *Ulva* spp.*)*<br>2) 3-5 months (e.g. *Cystoseira compressa*)<br>3) 6-11 months (e.g. *Peyssonnelia*, *Phyllariopsis*)<br>4) 1 year (e.g. *Halimeda)*<br>5) 2 years (e.g. most *Cystoseira*)<br>6) 2-5 years (e.g. *Cystoseira spinosa*)<br>7) > 5 years (e.g. *Corallium rubrum*)
11) Potential of asexual reproduction	Ordinal	2	1) No (e.g. *Halocynthia papillosa*)<br>2) Yes (e.g. colonial invertebrates*, Chondrosia reniformis)*
12) Growth rates	Ordinal	5	1) Extreme slow (<1 cm/year) (e.g. *Corallium rubrum*)<br>2) Slow (1 cm/ year) (e.g. *Cladocora, Ircina oros, Paramuricea, Lithophyllum stictaeforme)*<br>3) Moderate (>1 cm/year) (e.g. *Myriapora, Astroides)*<br>4) High (5-10 cm/year) (e.g. *Padina, Dictyota*, *Cystoseira balearica*)<br>5) Very high (>10 cm/year) (e.g. *Ulva, Cladophora vagabunda)*
13) Physical defenses/ Calcification	Categorical	5	a) Without (e.g. *Cystoseira, Sargassum, Ulva*)<br>b) Non-calcareous spicules (e.g. demosponges)<br>c) Calcareous spicules and sclerites (e.g. calcareous sponges, gorgonians)<br>d) External Carbonate (e.g. *Peyssonnelia harveyana*)<br>e) Carbonate with discontinuities (e.g. *Padina, Halimeda, Haliptilon, Corallina, Tricleocarpa*)<br>f) Continuous Carbonate (e.g. *Peyssonnelia rosa-marina, Lithophyllum*, corals, calcareous bryozoans)
14) Chemical defenses	Ordinal	2	1) No (e.g. *Ulva* spp.)<br>2) Yes (e.g. *Asparagopsis* spp., Crambe)
15) Motility	Ordinal	2	1) Sessile (e.g. algae and most invertebrates*)*<br>2) Vagile (e.g. *Paracentrotus lividus*)

Supplementary Table 3. Taxonomic and functional β-diversity comparisons among pH zones. Values show β-diversity, its two components (turnover and nestedness-resultant), and relative contribution of turnover to β-diversity (%).

	**Taxonomic**		**Functional**	
	Ambient pH	Low pH	Ambient pH	Low pH
**β-diversity**				
Low pH	0.48		0.17	
Extreme low pH	0.82	0.69	0.98	0.97
**Turnover**				
Low pH	0.40		0.10	
Extreme low pH	0.62	0.43	0.15	0.07
**Nestedness-resultant**				
Low pH	0.08		0.07	
Extreme low pH	0.20	0.26	0.82	0.90
**Turnover/ β-diversity (%)**				
Low pH	83		58	
Extreme low pH	75	62	15	7

Supplementary Table 4. Description of the reduced number of categories for each trait for the sensitivity analyses.

Trait	Trait Type	N	Categories (in parenthesis the original category)
1) Morphological form	Categorical	3	a) encrusting and boring, calcareous or not (a, d, e)<br>b) Filaments, erect or not; sheets, cylinders or blades, divided or not (b, c, f, g, h, m)<br>c) Massive form (i, j, k, l)
2) Solitary-Colonial	Ordinal	2	1) Solitary (1)<br>2) Colonial (2; 3)
3) Maximum longevity	Ordinal	2	1) Less than 1 year (1, 2)<br>2) More than 1 year (3, 4 ,5, 6; 7)
4) Growth rates	Ordinal	2	1) Extreme slow and slow (1, 2)<br>2) Moderate, high and very high (3, 4, 5)
5) Size (Height)	Ordinal	2	1) Up to 10 mm (1, 2)<br>2) >10- 200 mm (3, 4, 5, 6)
6) Size (Width)	Ordinal	2	1) Up to 1 mm (1, 2)<br>2) >1- 50 mm (3, 4, 5, 6)
7) Epibiosis	Ordinal	2	1) Obligate and Facultative (1, 2)<br>2) Never (3)
8) Energetic resource	Ordinal	2	1) Photosynthetic autotroph (1)<br>2) Mostly heterotroph (2, 3)
9) Major photosynthetic pigments	Categorical	2	a) No (autotroph) (a)<br>b) Chlorophyll and bacteriochlorophyll (b, c ,d, e, f, h, g)
10) Feeding	Categorical	3	a) No (a)<br>b) Filter feeders (b,c,d)<br>c) Herbivores (e) and carnivors (f)
11) Age at reproductive maturity	Ordinal	2	1) Less than 1 year (1, 2, 3 ,4)<br>2) more than 1 year (5, 6, 7)
12) Potential of asexual reproduction	Ordinal	2	1) No<br>2) Yes
13) Physical defenses/ Calcification	Categorical	2	a) Whithout and/or non-calcareous spicules (a, b)<br>b) With calcareous structures (c, d, e, f)
14) Chemical defenses	Ordinal	2	1) Non (1)<br>2) Yes (2)
15) Motility	Ordinal	2	1) Sessile (1)<br>2) Vagile (2)

Supplementary Table 5. Relative abundance (total sum) of functional trait categories (%) among pH zones.

Trait	Categories	Ambient pH	Low pH	Extreme Low pH
1) Morphological form	a) Boring	0.16	0	0
	b) Filaments	18.3	18.1	16.3
	c) Stolonial	10.0	8.9	1.3
	d) Encrusting	24.2	24.9	61.1
	e) Encrusting, leaf-like, with blades	7.0	6.5	0
	f) Foliose erect thallus, Sheets/blades	15.5	14.2	14.9
	g) Coarsely branched	7.0	19.1	5.7
	h) Articulated	13.0	4.4	0
	i) Cup-like	0.04	0	0
	j) Massive encrusting (height< radius)	1.4	1.9	0
	k) Massive hemispheric (height= radius)	3.3	1.9	0.1
	l) Massive-erect (height> radius	0.03	0.05	0.00
	m) Tree-like	0	0	0.6
2) Solitary-Colonial	1) Solitary	78.7	79.3	84.2
	2) Gregarious	16.2	14.9	15.8
	3) Colonial	5.1	5.8	0
3) Max. longevity	1) weeks	6.5	6.6	0
	2) 3-11 months	36.0	26.2	30.8
	3) 1 year	12.5	26.5	3.8
	4) 2 years	23.3	17.2	3.8
	5) 5 years	8.3	5.5	19.3
	6) 10 years -20 years	13.0	17.8	42.3
	7) > 20 years	0.4	0.2	0
4) Size (Height)	1) up to 1 mm	15.7	13.0	0.1
	2) 1-10 mm	22.5	25.3	75.7
	3) 10-50 mm	29.7	19.7	3.4
	4) 50-200 mm	32.2	41.9	20.3
	5) 200-500 mm	0	0	0.6
5) Size (Width)	2) 0.1-1 mm	0.5	0.1	0.1
	3) 1-10 mm	26.2	20.4	15.5
	4) 10-50 mm	57.9	61.1	24.0
	5) 50-200 mm	15.0	18.1	60.5
	6) >200 mm	0.4	0.2	0
6) Epibiosis	1) Obligate	1.6	2.7	0.1
	2) Facultative	57.2	55.3	34.2
	3) Never	41.2	42.0	65.8
7) Energetic resource	1) Photosynthetic autotroph	86.1	86.5	100.0
	2) Photo-heterotroph	0.6	0.3	0
	3) Heterotroph	13.3	13.2	0

Trait	Categories	Ambient pH	Low pH	Extreme Low pH
8) Photosynthetic pigments	a) No	13.3	13.2	0
	b) Chl a, Chl b, Beta-carotene, Xanthophyll	24.1	22.8	3.1
	c) Chl a, Xanthophyll /Fucoxanthin, Chl c1+c2	26.0	36.6	33.1
	d) Chla, Chlorophyll c2, Peridinin	0.2	0	0
	e) Chl a, Phycocyanin, Phycoerythrin	36.0	27.1	49.8
	f) Chla, Phycocyanin(Cyanobacteria)	0.5	0.3	0
	h) Mixture of a), b),c), e) (e.g. turf)	0.0	0.1	14.1
9) Feeding	a) No (autotroph)	86.1	86.5	100.0
	b) Active filter feeders with cilia	9.6	9.7	0
	c) Active filter feeders by pumping	4.0	3.8	0
	d) Passive filter feeders	0.2	0	0
	e) Herbivores/Grazers	0.1	0	0
10) Age reproductive maturity	1) weeks	7.6	5.0	0.4
	2) 3-5 months	38.7	42.5	19.5
	3) 6-11 months	28.8	27.7	18.3
	4) 1 year	22.3	21.2	61.8
	5) 2 years	0.3	0.1	0
	6) 2-5 years	2.4	3.4	0
11) Asexual reproduction	1) No	41.3	50.3	21.0
	2) Yes	58.7	49.7	79.0
12) Growth rates	2) Slow (1 cm/ year)	11.4	17.5	61.0
	3) Moderate (>1 cm/year)	36.9	23.0	2.6
	4) High (5-10 cm/year)	43.9	52.2	36.0
	5) Very high (>10 cm/year)	7.8	7.3	0.4
13) Calcification	a) Without	58.5	78.3	99.9
	b) Non-calcareous spicules	2.6	3.2	0
	c) Calcareous spicules and sclerites	0.9	0.4	0
	e) Carbonate with discontinuities	15.3	4.9	0
	f) Continuous Carbonate	22.7	13.2	0.1
14) Chemical defenses	1) No	95.9	93.9	100
	2) Yes	4.1	6.1	0
15) Motility	1) Sessile	99.9	100	100
	2) Vagile	0.1	0	0

Supplementary Note 1. Ecological relevance of functional traits

The traits were selected to describe complementary facets of ecology of benthic organisms that determine their response to disturbance and function in ecosystems. We coded the biological traits of the 72 taxa mainly based on the expertise of the team members (E. Ballesteros for algae and invertebrates and N. Teixidó for invertebrates), and other experts (sponges: E. Cebrian, S. de Caralt, M.J. Uriz, J. Vacelet and tunicates: X. Turón), identification guides (http://www.algaebase.org/, http://doris.ffessm.fr/, http://corspecies.medrecover.org/) and literature. See Table S2 for categories and trait codes.

Morphological form

The growth form (morphology) of a benthic species determine its competitive ability for space and light, through maximizing photosynthetic production, disturbance (both biotic and abiotic), and food supply[1]. Growth form was coded as a categorical trait, with 13 categories: from boring species to tree-like forms.

Solitary-Colonial life histories

Solitary-Colonial strategies, like morphology, have an important adaptive significance regarding competition for space[1]. Solitary forms are distinct individuals whereas colonial forms are built up of modules/ polyps or zooids. We also include the gregarious strategy, consisting in groups of solitary individuals. This trait was coded as ordinal with 3 categories: solitary, gregarious, and colonial.

Maximum longevity

Longevity of species indicates resistance to repeated disturbances as well as nutrient storage [2]. Longevity was coded as ordinal along a continuum, with 7 categories: from weeks to more than 20 years.

Growth rates

Growth rate, like longevity, is a prominent indicator of the dynamics of benthic communities, environmental stress and disturbance regimes. Typically, long-lived species exhibit slow growth rates whereas short-lived species grows faster. Growth rate was coded as ordinal along a continuum, with 5 categories: from extreme slow (<1 cm/year) to very high (>100 cm/year).

Size (height and width)

Size (height and width) is associated with energy demand, competition for space, and resistance to predation. Height and width were coded as ordinal along a continuum, both with 6 categories: from extreme small (up to 1 mm) to very large (> 500 mm for height, and > 200 mm for width, respectively).

Epibiosis

Some benthic species depend on other species to attach. Epibiosis was coded as ordinal along a continuum, with 3 categories: obligate, facultative, and never.

Energetic resource, major photosynthetic pigments and feeding

Energetic resources, feeding and major photosynthetic pigments determines species impact on ecosystem functioning through tropic interactions and on nutrient cycling. Energetic resource was coded as ordinal, with 3 categories: photosynthetic autotroph, photo-heterotroph, and heterotroph. Feeding was coded as categorical, with 6 categories: from no-feeding to carnivores. Major photosynthetic pigments were coded as categorical, with 8 categories: from absence of pigments to a mixture of different pigments.

Age at reproductive maturity

Age at reproductive maturity is an important component to characterize population dynamics of communities. Age at reproductive maturity was coded as ordinal, with 7 categories along a continuum: from weeks to more than 5 years.

Potential of asexual reproduction

Potential of asexual reproduction (as fragmentation, regrowth of existing colonies, internal gemmules, and external propagules) has implications regarding the costs and benefits of energy allocation under different regimes of mortality and the recovery of populations. Potential of asexual reproduction was coded as a binary trait with 2 categories: no and yes.

Physical defenses (calcification)

Physical defenses have a primary role in defense against predation (e.g. from sea urchins and/or fishes). Physical defense was coded as categorical, with 5 categories: from without to continuous carbonate shells and skeletons.

Chemical defenses

Chemical defenses have an important role for predation deterrence, prevention of fouling, inhibition of overgrowth, and protection from ultraviolet radiation[3]. Chemical defense was coded as a binary trait with 2 categories: no and yes.

Mobility

Benthic communities include sessile and vagile species. Mobility was coded as ordinal trait with 2 categories: sessile and vagile.

Supplementary References

1. Jackson, J. Morphological strategies of sessile animals. in *Biology and Systematics of Colonial Organisms* (eds. Larwood, G. & Rosen, B.) 499–555 (Academic Press, 1979).
2. Teixidó, N., Garrabou, J. & Harmelin, J. G. Low dynamics, high longevity and persistence of sessile structural species dwelling on mediterranean coralligenous outcrops. *PLoS One* **6,** e23744 (2011).
3. Pawlik, J. R. Marine Invertebrate Chemical Defenses. *Chemical Reviews* **93,** 1911–1922 (1993).
